# Supplementary material for: Biocontrol of mushroom crop mycoparasites by novel Bacillus velezensis strains
Source: Appl Microbiol Biotechnol. 2026 Jul 6;110:259. doi: 10.1007/s00253-026-13938-3 (PMC13522302; doi:10.1007/s00253-026-13938-3)
Supplement: Supplementary file 1 [file 253_2026_13938_MOESM1_ESM.pdf]

Supplementary Material for ‘Biocontrol of mushroom crop mycoparasites by novel *Bacillus velezensis* strains’

Applied Microbiology and Biotechnology

William Kay[‡,1]; Jaime Carrasco[‡,1,2,3,4*]; Souvik Kusari[5,6]: Marjon Krijger[7]; M. José Carpio[4,8]; Thomas Barnes[1]; M. Sonia Rodríguez Cruz[4]; Jan van der Wolf[7]; Till Bebenroth[6]; Gail M. Preston*[1]

[1]University of Oxford, Department of Biology, South Parks Road, Oxford, OX1 3RB, United Kingdom.

[2]IRIAF – Agri-food and Forestry Regional Research and Development Centre, CIAF - Agroforestry Research Centre, Carretera Toledo-Cuenca km 174, Cuenca 16194, Spain.

[3]Centro Tecnológico de Investigación del Champiñón de La Rioja (CTICH), Autol, Spain.

[4]Institute of Natural Resources and Agrobiology of Salamanca, Cordel de Merinas 40-52, 37008 Salamanca, Spain.

[5]Department of Genetics, Institute of Biology and Ecology, Faculty of Science, Pavol Jozef Šafárik University in Košice, Mánesova 23, 04154 Košice, Slovakia.

[6]Center for Mass Spectrometry, Faculty of Chemistry and Chemical Biology, Technische Universität Dortmund, 44227 Dortmund, Germany.

[7]Wageningen University and Research, Biointeractions & Plant Health, 6700 AA Wageningen, the Netherlands;

[8]Institute of Agricultural Sciences, ICA-CSIC, Serrano 115 bis, 28006 Madrid, Spain

[‡]These authors are joint first authors

*Corresponding authors: jcarrasco@jccm.es (JC); gail.preston@biology.ox.ac.uk (GP)

Supplementary Methods

Supplementary Methods S1: Specificity of the TaqMan assays: The specificity of the assays was tested using genomic DNA of 27 strains. For most strains 0.5 ng of purified DNA was used, but for some strains a suspension of 10^9- 10^{10} cells/mL was boiled for 10 min in 150 µL MilliQ water (MQ) prior to testing. For each TaqMan assay 2 µL of DNA or the boiled suspensions was mixed with 2 µL reaction mix containing PerfeCTa multiplex qPCR ToughMix 5x (Quantabio, Beverly, USA), 100 nM probe and 300 nM of each forward and reverse primer. The reactions were performed in a 12K Flex QuantStudio or a QuantStudio 5 Real-Time PCR system (Applied Biosystems, Thermo Fisher Scientific, Waltham, MA, USA) using the following conditions: 95 °C for 2 min; 40 cycles of 95 °C for 15 s followed by 60 °C for 60 s. Analysis of the data was done by automatic threshold calculation within the Applied Biosystems software. A Ct value ≤ 35 was considered positive.

The specificity and usability were further tested using constituents of casing soils or casing soils of various compositions. DNA was extracted from 10 g of material using the DNeasy PowerMax Soil kit (QIAGEN N.V., Venlo, The Netherlands) according to the manufacturer's protocol. TaqMan assays were performed as described above with 2 µL DNA.

Supplementary Methods S2: Triplex TaqMan design: The two simplex assays for the target bacteria were combined with an assay that quantifies *Xanthomonas campestris* pv. *campestris* (Xcc) into a triplex TaqMan (Köhl et al. 2011; Taparia et al. 2020). For each group of biostimulants, a triplex TaqMan assay was designed with two target-specific assays, each with its own dye (Cy5 or FAM) and an amplification and extraction control (*X. campestris*), labelled with HEX. The triplex TaqMan was performed using the same PCR conditions and materials as the simplex tests, with 100 nM probe and 300 nM of each primer in a total volume of 25 µL.

Supplementary Methods S3: Limit of detection of the TaqMan assays: The limit of detection for the triplex assays designed for *B. velezensis* strains CM19, CM5 and CM35 (Supplementary Table S7) was determined in two casing soils, i.e. in peat and steamed spent casing soil. 100 µL of a ten-fold serial dilution of 3-days old cultures of the strains in Ringers in a PowerBead Pro tube (QIAGEN GmbH, Hilden, Germany) was mixed with 250 mg of the casings ($4 \times 10^7 – 4 \times 10^1$ cells g^{-1} of casing soil). Casing soils with 100 µL Ringers without supplemented bacteria served as a control. A volume of 100 µL of a suspension of *X. campestris* (Xcc) of 10^6 cell mL^{-1} was added to each sample as extraction and amplification

control. The soils were subsequently freeze dried overnight and shaken for 2 times 90 seconds in a paint shaker. DNA was extracted using a DNeasy Power Soil Pro Kit (QIAGEN GmbH, Hilden, Germany) according to the manufacturer's instructions. The triplex TaqMan assay was executed as described above with 0.75 µL of each primer (10 µM) and 0.75 µL of each probe (10 µM), labelled with FAM, Cy5 (for target specific probes) or HEX (for Xcc).

Supplementary Tables

Supplementary Table S1: Cultivable bacterial isolates from the peat casing layer and basidiomes.

Strain code	Origin (isolated from)[1]	Strain (based on sequencing of 16S rRNA)[2]
CM5	CM	*Bacillus velezensis* (HQ831396.1) - 98.76%
CM19	CM	*B. velezensis* (MT184842.1) - 83.25%
CM35	CM	*B. velezensis* (MF581451.1) - 99.43%
B10	B	*Pantoea* sp. (MK398041.1) - 82.66%
B13	B	*Serratia* sp. (MT623418.1) - 98.21%
B18	B	*Staphylococcus fleurettii* (MN758802.1) - 98.43%
B19	B	*Staphylococcus* sp. (KT029134.1) - 99.25%
B9A	B	*Serratia* sp. (KF791514.1) - 98.10%
B9B	B	*Vibrio ruber* (MT422024.1) - 84.38%
B9C	B	*Pseudochrobactrum asaccharolyticum* (KF263562.1) - 99.21%
B11W	B	*Staphylococcus fleurettii* (MK015775.1) - 99.30%
B11Y	B	*Arthrobacter* sp. (MG874752.1) - 99.35%
B14A	B	*Serratia marcescens* (MT645673.1) - 98.21%
B14B	B	*Paenochrobactrum* sp. (JF804769.1) - 98.92%
B15W	B	*Staphylococcaceae* bacterium (AY841364.1) - 77.00%
B15Y	B	*Glutamicibacter* sp. (MW627371.1) - 100%
B16	B	*Pseudomonas* sp. (MK949377.1) - 98.70%
B17A	B	*Klebsiella oxytoca* (MF144451.1) - 98.90%
B17B	B	*Pseudochrobactrum* sp. (CP075349.1) - 82.13%
B20A	B	*Bacillus* sp. (KM242479.1) - 95.80%
B20B	B	Uncultured bacterium (DQ817818.1) - 85.82%
B21A	B	*Glutamicibacter mishrai* (CP032549.1) - 99%
B21B	B	*Staphylococcus* sp. (MT516468.1) - 92.47%

B21C	B	*Pseudomonas putida* 1616X1 -98%
B22A	B	*Pseudomonas putida* JQ581 92%
B22B	B	*Staphylococcus saprophyticus* poll1 - 96.69%
CM1	CM	*Pseudomonas* sp. (JF430830.1) - 79.95%
CM2	CM	*Pseudomonas* sp. (MN231751.1) - 98.25%
CM3	CM	*Serratia* sp. strain AXJ-M - 98.62
CM4	CM	*Serratia* sp. (MN928759.1) - 94.85%
CM6	CM	*Serratia* sp. (MT472080.1) - 94.93%
CM7	CM	*Serratia* sp. (MT472080.1) - 96.18%
CM10	CM	*Serratia* sp. (MT472080.1) - 94.21%
CM11	CM	*Bacillus* sp. (DQ365574.1) - 78.31%
CM12	CM	*Chryseobacterium* sp. (KX928035.1) - 80.14%
CM13	CM	No significant similarity found
CM14	CM	*Pseudomonas* sp. (KF263605.1) - 91.85%
CM15	CM	*Brevundimonas* sp. (MK110476.1)
CM16	CM	Uncultured bacterium (KX507468.1) - 73.16%
CM17	CM	*Comamomas* sp. (KC294078.1) - 99.33%
CM18	CM	*B. velezensis* LA-33 - 95%
CM20	CM	*B. velezensis* WC-14 - 95.15%
CM21	CM	*B. velezensis* YJ11-1-4 = 90.71%
CM22	CM	*Pseudomonas putida* (MN795707.1) - 88.42%
CM23	CM	*B. licheniformis* (MN756663.1) - 97.04%
CM25	CM	*Pseudomonas* sp. (JX678920.1) - 78.40%
CM26	CM	*Bacillus* sp. (MN989043.1) - 98.43%
CM27	CM	*Acidovorax radicis* IMCC34918 - 94.59%
CM30	CM	*Pseudomonas* sp. (MT568555.1) - 98.55%
CM31	CM	*Rhodococcus* sp. CP5 - 86.34%
CM32	CM	*Glutamicibacter halophytocola* (MT533909.1) - 96.24%
CM33	CM	*Pseudomonas* sp. (KF202578.1) - 78.63%
CM34	CM	*Serratia* sp. (MT472080.1) - 95.33%
CM36	CM	*Serratia* sp. strain TC/03 - 96.44%
CM37	CM	*Serratia* sp. (MT472080.1) - 95.29%

CM38	CM	*Klebsiella* sp. (MK209044.1) - 87.85%
CM39	CM	*Pseudomonas frederiksbergensis* (MG561792.1) - 74.47%
CM40	CM	*Pseudomonas* sp. - Uncultured bacterium (GQ101186.1) - 74.39%
CM41	CM	*Pseudochrobactrum* sp. (MK095772.1) - 81.12%
CM42	CM	*Stenotrophomonas* sp. (MT258986.1) - 84.93%
CM43	CM	*Glutamicibacter* sp. (MK841094.1) - 97.99%
EM1	B	*Bacillus algicola* (MN746261.1) - 96.45%
EM2	B	*Bacillus* sp. (FN646629.1) - 97.80%
EM3	B	*Escherichia coli* (CP044298.1) - 89.26%
EM4	B	*Kluyvera intermedia* (MT081698.1) - 93.37%
EM5	B	*B. velezensis* (MN272328.1) - 99.28%
EM6	B	*Staphylococcus succinus* (KX348384.1) - 98.62%
EM7	B	*Bacillus firmus* (KY316472.1) - 99.33%
EM8	B	*Bacillus licheniformis* (JX041900.1) - 75.69%
EM9	B	Uncultured *Pseudomonas* sp. (HQ616313.1) - 79.01%
EM10	B	*Stenotrophomonas* sp. (KC853134.1) - 83.03%
EM11	B	*Pseudomonas fluorescens* (JX484838.1) - 84.65%
EM12	B	*Pseudomonas* sp. P.f-9103 (KM032182.1) - 90.31%
EM15S	B	*Stenotrophomonas rhizophila* (JQ670922.1) - 89.31%
EM15L	B	*Rhodococcus* sp. (MH043947.1) - 95.93%
EM16	B	*Lampropedia* sp. BRTC-3 (KU612256.1) - 97.10%
EM17	B	*Rhodococcus* sp. (JF742932.1) - 75.57%
EM18	B	*Enterobacteriaceae* bacterium (AB275371.1) - 79.66%
EM19	B	*Pseudomonas* sp. SB11-07 (GU595316.1) - 82.58%
EM21	B	*Pseudomonas* sp. (KX264924.1) - 98.47%
EM22	B	*Pseudomonas* sp. (JN201547.1) - 98.19%
EM23	B	*Pseudomonas* sp. (KU510013.1) - 98.67%
EM24	B	*Ochrobactrum* sp. (MT271895.1) - 99.70%
EM25	B	*Pseudomonas* sp. (MW737403.1) - 98.11%
EM27	B	*Paracoccus* sp. (KY971629.1) - 99.38%
EM28	B	*Stenotrophomonas maltophilia* (DQ339644.1) - 90.60%
EM29	B	*Pseudomonas aeruginosa* (CP042269.1) - 92.67%

EM30	B	*Microbacterium* sp. (MT576542.1) - 98.51%
EM31	B	*Microbacterium* sp. (JQ977247.1) - 97.67%
EM32	B	*Serratia* sp. (AM231089.1) - 89.77%
EM33	B	*Rahnella inusitata* (MT555345.1)- 91.28%
EM34	B	*Bacillus* sp. (GQ199751.1) - 96.04%
EM35	B	*Staphylococcus* sp. (KR029198.1) - 97.27%
EM36	B	Uncultured bacterium-*Enterobacteria* (KP142371.1) - 72.48%
EM37	B	*Staphylococcus* sp. (MN103949.1) - 98.85%
EM38	B	*Microbacterium* sp. (MH130312.1) - 92.11%
EM39	B	*Bacillus amyloliquefaciens* (KJ639041.1) - 84.50%
EM41	B	*Citrobacter* sp. SI-GU-1B (KP322786.1) - 78.91%
EM42	B	*Pseudomonas fluorescens* (FJ787327.1) - 84.16%
EM43	B	*Chryseobacterium* sp. (DQ530065.1) - 91.17%
EM44	B	*Pseudomonas* sp. (MK371440.1) - 83.87%
EM45	B	*Pseudomonas* sp.185 - 97.62%
EM46	B	*Pseudomonas koreensis* (MW405680.1) -88.78%
EM47	B	*Pseudomonas* sp. (MW433970.1) - 97.37%
EM48	B	*Pseudomonas mosselii* (KY426038.1) - 97.48%
EM49	B	*Exiguobacterium* sp. (MT611261.1) - 97.89%
EM13	B	*Chryseobacterium* sp. (KX079844.1) - 91.73%
EM14	B	*Enterobacter* sp. (FJ587228.1) - 79.77%
EM20	B	*Chryseobacterium* sp. (KX079844.1) - 87.50%
EM26	B	*Chryseobacterium* sp. (MG893574.1) - 90.68%
EM40	B	*Serratia marcescens* (EF635971.1) - 89.23%
CM8	CM	*Pseudomonas laurentiana* SeaQual_P_B70/W - 89%
CM9	CM	*Pseudomonas putida* (AF307869.1) - 99.16%
CM24	CM	*Pseudomonas* sp. (KM391420.1) - 96.38%
CM28	CM	Uncultured *Pseudomonas* sp. (EU029490.1) - 82.52%
CM29	CM	*Serratia grimesii* (DQ481467.1) - 84.32%
CM44	CM	Uncultured *Pseudomonas* sp. (HM011771.1) - 88.41%
CM45	CM	*Glutamicibacter* sp. (MK847918.1) - 98.04%
CM46	CM	*Citrobacter werkmanii* (LR699014.1) - 83.62%

CM47	CM	*Ochrobactrum* sp. (KY678891.1) - 89.09%
CM48	CM	*Micrococcus endophyticus* (MT214326.1) - 100%
CM49	CM	*Streptomyces* sp. (KF650632.1) - 76.03%
CM50	CM	*Pseudochrobactrum* sp. (KF937789.1) - 83.43%
CM51	CM	*Citrobacter freundii* (MW940831.1) - 79.31%
CM52	CM	*Alcaligenaceae* bacterium (MH593839.1) - 89.41%
CM53	CM	*Rhodococcus* sp. (MT631993.1) - 100%
CM54	CM	*Brevundimonas* sp. (MF111489.1) - 96.57%
CM56	CM	*Pseudomonas plecoglossicida* (KF261039.1) - 100%
CM57	CM	*Pseudomonas* sp. WB4.4-63 (AM934697.1) - 84.82%
CM58	CM	No significant similarity found
CM59	CM	Uncultured bacterium (KP156579.1) - 75.83%
CM60	CM	*Brevundimonas diminuta* (KC152996.1) - 75.27%
CM61	CM	*Brevundimonas staleyi* EB109 - 88.22%
CM62	CM	*Enterobacter* sp. (MT557025.1) - 75.00%
CM63	CM	*Bacillus oceanisediminis* SH89 - 91.70%
CM55	CM	*Pseudomonas paralactis* BF388 - 94.97%

[1]The strains were isolated either from casing material (CM) or adult basidiomes (B). [2]BLASTn (blast.ncbi.nlm.nih.gov) was used to classify the microbes based on their 16s rRNA sequences.

Supplementary Table S2: Media employed to isolate and culture bacteria from the casing and *A. bisporus* basidiomes.

Media	Recipe
Agar-casing-LBA	150 g of dried and milled fresh casing are autoclaved with approx. 800 mL of distilled water at 121ºC and 1 at. The supernatant is filtered through a double layer of muslin to remove the bulk of the substrate, and 10 g of tryptone (Cultimed, Barcelona, Spain Panreac #403682, Barcelona, Spain), 5 g of yeast extract (Cultimed, Barcelona, Spain #A3732), + 10 g NaCl (Thermo Fisher Scientific, Waltham, Beverly, MA, USA #207790010) and 15 g of bacteriological agar (Cultimed, Barcelona, Spain #402302) are added to the supernatant liquid up to 1 L with distilled water.
Agar-compost	50 g of dried (60ºC for 72h in a forced air furnace) and ground to powder (using electrical crusher) phase II compost for *A. bisporus* are autoclaved with approx. 800 mL of distilled water at 121ºC and 1 at. The supernatant is filtered through a double layer of muslin to remove the bulk of the substrate, and 15 g of bacteriological agar (Cultimed, Barcelona, Spain #402302) is added up to 1 L with distilled water. The dried phase II powder is stored in a zip bag at room temperature for max. 6 months.

King´s agar + cfc	Selective medium for *Pseudomonas*: Peptone-protease No.3 (Thermo Fisher Scientific, Waltham, MA, USA #211693), 20 g L^{-1}; K_2HPO_4 (Thermo Scientific #215472500), 1.5 g L^{-1}; $MgSO_4$ (Thermo Fisher Scientific, Waltham, MA, USA #A14491.0I), 1.5 g L^{-1}; Bacteriological agar, 15 g L^{-1} (Cultimed #402302); Glycerol (Thermo Fisher Scientific, Waltham, MA, USA #A16205.AP), 10 mL L^{-1}, plus CFC *Pseudomonas* supplement (Thermo Fisher Scientific, Waltham, MA, USA #SR0103E) (content of a vial for 500 mL of medium): cetrimide (5 mg); Fusionic acid (5 mg); Cephalosporin (25 mg).
LBA	10 Tryptone (Cultimed, Barcelona, Spain) + 5 g Yeast extract (Cultimed, Barcelona, Spain) + 10 g NaCl (Thermo Fisher Scientific, Waltham, MA, USA) + 15 g Bacteriological agar (Cultimed, Barcelona, Spain) for 1L;
TSA	Tryptone soya agar was prepared according to the manufacturer's instructions (Cultimed, Barcelona, Spain #416106)
Hicrome™ b. agar	Selective medium was prepared according to the manufacturer's instructions (HiMedia Laboratories Pvt. Ltd., Mumbai, India).
Pbs electroporation buffer	PEB: 272 mM sucrose, 1 mM $MgCl_2 \cdot 6H_2O$, 7 mM phosphate buffer, pH 7.4)

Supplementary Table S3: Quality assessment of *B. velezensis* genomes using QUAST.

Statistics without reference[1]	064048E_CM5	064049E_CM35	064050E_EM5	064051E_EM39
# contigs	17	16	17	11
# contigs (>= 0 bp)	46	32	32	48
# contigs (>= 1000 bp)	13	13	14	10
# contigs (>= 5000 bp)	8	8	9	7
# contigs (>= 10000 bp)	8	8	9	7
# contigs (>= 25000 bp)	7	7	8	6
# contigs (>= 50000 bp)	7	7	8	6
Largest contig	2233836	2233835	2233835	2061313
Total length	4203474	4202775	4202331	3891334
Total length (>= 0 bp)	4213899	4207978	4206541	3902642
Total length (>= 1000 bp)	4200517	4200358	4199939	3890670
Total length (>= 5000 bp)	4192636	4192415	4192079	3885132
Total length (>= 10000 bp)	4192636	4192415	4192079	3885132
Total length (>= 25000 bp)	4175744	4175523	4175187	3868234
Total length (>= 50000 bp)	4175744	4175523	4175187	3868234
N50	2233836	2233835	2233835	2061313

N75	488218	488218	488307	1012020
L50	1	1	1	1
L75	3	3	3	2
GC (%)	45.84	45.85	45.84	46.43
Mismatches				
# N's	0	0	0	0
# N's per 100 kbp	0	0	0	0

[1]All statistics are based on contigs of size >= 500 bp, unless otherwise noted (e.g., "# contigs (>= 0 bp)" and "Total length (>= 0 bp)" include all contig. Kbp = kilobase pairs. The 16S rDNA region was amplified using primers 27F/1492R (Stackebrandt and Goodfellow 1991)

Supplementary Table S4: Primers used in this study for the phylogenetic analysis of isolated *B. velezensis* strains.

Primer name	Sequence (5' - 3')	Ref.
16s_27F	AGAGTTTGATCCTGGCTCAG	Taguchi et al. (2008)
16s_1492R	GGTTACCTTGTTACGACTT	Taguchi et al. (2008)
rpoB24F	CGCATGATTTGAGGGG	Fajardo-Cavazos et al. (2018)
rpoB737R	GGCGGCTCTCCAGG	Fajardo-Cavazos et al. (2018)
tufGPF	ACGTTGACTGCCCAGGACAC	Draganic et al. (2017)
tufGPR	GATACCAGTTACGTCAGTTGTACGGA	Draganic et al. (2017)
WK_rpoD_F	CCACGAAACAGAAACAGAACTTAC	This paper
WK_rpoD_R	CTGCTCGGATGTCTCAATTTG	This paper
WK_pgk_2_F	CATCGACGTAAAAGGCAAAG	This paper
WK_pgk_2_R	CTACACCTGGAAGCTCTTTG	This paper
Wk_gyrB_2_F	GTATTAGAAGGTTTGGAAGCTGTTCG	This paper
WK_gyrB_2_R	CATTTCTCCAAGACCTTTATAACGC	This paper

Supplementary Table S5: Experimental blocks used in growth chamber crop trial

**Treatment**	**Code**	**Dose[1]**	**Infected (*Z. fungicola* 150/1)[2]**
**Control**	Control+	20 ml tap water	10^6 conidia m^{-2}
**Control**	Control-	20 ml tap water	-
***B. velezensis* CM5**	CM5	10^9 cfu m^{-2}	10^6 conidia m^{-2}
***B. velezensis* CM19**	CM19	10^9 cfu m^{-2}	10^6 conidia m^{-2}
***B. velezensis* CM35**	CM35	10^9 cfu m^{-2}	10^6 conidia m^{-2}

**Prochloraz-Mn**	PCL	$1\ g\ m^{-2}$	10^{6} conidia m^{-2}

[1]PCL was applied 4 days after casing, bacteria were applied 5 days after casing. cfu: colony forming unit. [2]Conidia were applied 10 days after casing.

Supplementary Table S6. Specificity of TaqMan assays developed for *B. velezensis* strains selected as biocontrol agents for *A. bisporus* crops.

**Species**	**[1]Strain No**	**Geographic origin**	**Yr of isolation**	**Isolated from:**	**[2]*B. velezensis* TaqMan (Ct)**	
					**2HP2.21**	**HP2.19**
Pseudomonas putida	PMS118R	New Zealand	1990	Casing - mushroom farms	ND	ND
Pseudomonas fluorescens	SBW25	United Kingdom	1989	*Beta vulgaris*	ND	ND
P. fluorescens	PMS232	United Kingdom	1989	*Beta vulgaris*	ND	ND
P. fluorescens	55				ND	ND
Pseudomonas sp.	B18	Spain	2017	Casing - mushroom farms (this study)	ND	ND
Pantoea sp.	B11	Spain	2017	Casing - mushroom farms (this study)	ND	ND
Serratia sp.	B4	Spain	2017	Casing - mushroom farms (this study)	ND	ND
B. velezensis	CM19	Spain	2017	Casing - mushroom farms (this study)	24.2	23.4
B. velezensis	CM5	Spain	2017	Casing - mushroom farms (this study)	24.2	24
B. velezensis	CM35	Spain	2017	Casing - mushroom farms (this study)	23.7	23.1
Bacillus sp.	B20A	Spain	2017	Casing - mushroom farms (this study)	ND	ND
Bacillus sp.	CM11	Spain	2017	Casing - mushroom farms (this study)	ND	ND
B. velezensis	CM18	Spain	2017	Casing - mushroom farms (this study)	15	14.1
B. velezensis	CM20	Spain	2017	Casing - mushroom farms (this study)	15.8	14.9
B. velezensis	CM21	Spain	2017	Casing - mushroom farms (this study)	15.5	14.1
Bacillus sp.	CM26	Spain	2017	Casing - mushroom farms (this study)	ND	ND

Bacillus algicola	EM1	Spain	2020	Mushroom endophyte (this study)	ND	ND
Bacillus sp.	EM2	Spain	2020	Mushroom endophyte (this study)	ND	ND
B. velezensis	EM5	Spain	2020	Mushroom endophyte (this study)	15.1	14.3
Bacillus firmus	EM7	Spain	2020	Mushroom endophyte (this study)	ND	ND
Bacillus licheniformis	EM8	Spain	2020	Mushroom endophyte (this study)	ND	ND
Bacillus sp.	EM34	Spain	2020	Mushroom endophyte (this study)	ND	ND
B. velezensis	EM39	Spain	2020	Mushroom endophyte (this study)	ND	ND
B. subtilis	KDSA	-	-	-	ND	ND
B. thuringiensis	407 cry-	France	198?	Isolated as a lepidopteran-active strain (acrystalliferous derivative)	ND	ND
B. subtilis	168	USA	194?	Model organism	ND	ND
	[3]MQ				ND	ND

[1] Target strains are highlighted in green cells. [2] Per strain, two TaqMan assays were used (2HP2.21 and HP2.19). All probes were labelled with FAM (6-carboxyfluorescein, green-fluorescent dye). ND = not detected after 40 cycles. Ct (Cycle threshold): value is the number of PCR cycles required for the fluorescent signal to cross a certain threshold, indicating detectable levels of the target nucleic acid. [3]MQ= Molecular quantification.

Supplementary Table S7: TaqMan primers and probes for *B. velezensis* (strains CM5, CM19; CM35) and for the amplification and extraction control *X. campestris* pv. *campestris*.

	**Species**	***B. velezensis***		***X. campestris* pv. *campestris***
	**Strain No.**	**CM19; CM5; CM35**		**Extraction control**
	**Target gene**	**Hypothetical protein**	**Hypothetical protein**	**Unknown**
**Forward primer**	Name	*B. velezensis*_HP2.21_set2_F	*B. velezensis*_HP2.19_set3_F	XccF
	Sequence (5'-3')	CCCTGAACATAG GGTGTATGATG	CATTATTCAGACAC AGATGCTCTT	GTGCATAGGCCAC GATGTTG
**Reverse Primer**	Name	*B. velezensis* _HP2.21_set2_R	*B. velezensis* _HP2.19_set3_R	XccR
	Sequence (5'-3')	ACAGCAATTGGT TGGAAGAAAC	TGAAGACATTATTGA ATGCTTACCC	CGGATGCAGAGCG TCTTACA
**Probe**	Name	*B. velezensis* _HP2.21_P	*B. velezensis* _HP2.19_P	XccP

	Sequence (5'-3')	TTTGGAATCCCAC GTTCGTTTACA	ACCTGCTGACTGTTT GCAAACTCT	CAAGCGATGTACT GCGGCCGTC
	Dye[1]	FAM	Dy5	HEX

[1]Label in Triplex assay.

Supplementary Table S8: Results of antimicrobial confrontation assays of cultivable bacterial microbiome from the peat casing layer and positive control strains NZ011, NZI7 and NCPPB2192 against 4 major fungal parasites of *A. bisporus*: (i) green mould/*Trichoderma aggressivum* TAV1 (Ta), (ii) wet bubble/*Hypomyces perniciosus* M25 (Hp), (iii) dry bubble/*Zarea fungicola* 150/1 (Zf), and (iv) cobweb/*Hypomyces odoratus* CM13900 (Ho). Percentages indicate the proportion of experimental replicates in which a confrontation between the bacterial strain and fungal strain was observed.

**Code**	**Strain (Seq. result 16S rRNA)**	**Ta**	**Hp**	**Zf**	**Ho**
CM5	*B. velezensis* CM5	100%	100%	100%	100%
CM19	*B. velezensis* CM19	100%	100%	100%	100%
CM35	*B. velezensis* CM35	100%	100%	100%	100%
NZ011	*Pseudomonas fluorescens* NZ011	100%	100%	100%	100%
NZI7	*Pseudomonas fluorescens* NZI7	100%	100%	100%	100%
NCPPB2192	*Pseudomonas tolaasii* NCPPB 2192 (smooth)	100%	100%	0%	0%
B16	*Pseudomonas* sp. (MK949377.1) - 98.70%	0%	0%	0%	0%
CM1	*Pseudomonas* sp. (JF430830.1) - 79.95%	0%	0%	0%	0%
CM2	*Pseudomonas* sp. (MN231751.1) - 98.25%	0%	0%	0%	0%
CM11	*Bacillus* sp. (DQ365574.1) - 78.31%	0%	0%	0%	0%
CM14	*Pseudomonas* sp. (KF263605.1) - 91.85%	0%	0%	0%	0%
CM18	*B. velezensis* strain LA-33 - 95%	100%	100%	100%	100%
CM20	*B. velezensis* strain WC-14 - 95.15%	100%	100%	100%	100%
CM21	*B. velezensis* strain YJ11-1-4 = 90.71%	100%	100%	100%	100%
CM22	*Pseudomonas putida* (MN795707.1) - 88.42%	0%	0%	75%	0%
CM23	*Bacillus licheniformis* (MN756663.1) - 97.04%	0%	0%	0%	0%
CM25	*Pseudomonas* sp. (JX678920.1) - 78.40%	0%	0%	0%	0%
CM26	*Bacillus oceanisediminis* strain Xmb066 - 98.47%	0%	0%	0%	0%
CM30	*Pseudomonas* sp. (MT568555.1) - 98.55%	0%	0%	0%	0%
CM33	*Pseudomonas* sp. (KF202578.1) - 78.63%	100%	100%	100%	0%
CM39	*Pseudomonas frederiksbergensis* (MG561792.1) 74.47%	0%	0%	100%	0%
CM40	*Pseudomonas* sp. Uncultured (GQ101186.1) - 74.39%	0%	100%	0%	0%
EM5	*B. velezensis* (MN272328.1) - 99.28%	100%	100%	100%	100%
EM9	Uncultured *Pseudomonas* sp. (HQ616313.1) - 79.01%	0%	0%	0%	0%
EM11	*Pseudomonas fluorescens* (JX484838.1) - 84.65%	0%	0%	100%	0%

EM12	*Pseudomonas* sp. P.f-9103 (KM032182.1) - 90.31%	0%	0%	0%	0%
EM19	*Pseudomonas* sp. SB11-07 (GU595316.1) - 82.58%	0%	0%	100%	0%
EM21	*Pseudomonas* sp. (KX264924.1) - 98.47%	0%	0%	33%	0%
EM22	*Pseudomonas* sp. (JN201547.1) - 98.19%	0%	0%	0%	0%
EM23	*Pseudomonas* sp. (KU510013.1) - 98.67%	0%	0%	0%	0%
EM25	*Pseudomonas* sp. (MW737403.1) - 98.11%	0%	0%	0%	0%
EM29	*Pseudomonas aeruginosa* (CP042269.1) - 92.67%	0%	0%	0%	0%
EM39	*Bacillus amyloliquefaciens* (KJ639041.1) - 84.50%	100%	100%	75%	100%
EM42	*Pseudomonas fluorescens* (FJ787327.1) - 84.16%	0%	0%	0%	0%
EM44	*Pseudomonas* sp. (KX264924.1) - 98.47%	0%	0%	0%	0%
EM46	*Pseudomonas koreensis* (MW405680.1) -88.78%	100%	100%	100%	0%
EM47	*Pseudomonas* sp. (MW433970.1) - 97.37%	0%	0%	0%	0%
EM48	*Pseudomonas mosselii* (KY426038.1) - 97.48%	100%	100%	100%	0%
CM9	*Pseudomonas putida* (AF307869.1) - 99.16%	0%	0%	0%	0%
CM24	*Pseudomonas* sp. (KM391420.1) - 96.38%	0%	0%	0%	0%
CM28	Uncultured *Pseudomonas* sp. (EU029490.1) - 82.52%	100%	100%	100%	0%
CM44	Uncultured *Pseudomonas* sp. (HM011771.1) - 88.41%	0%	0%	0%	0%
CM56	*Pseudomonas plecoglossicida* (KF261039.1) - 100%	0%	100%	100%	0%
CM57	*Pseudomonas* sp. WB4.4-63 (AM934697.1) - 84.82%	0%	0%	0%	0%
CM64	*B. velezensis* QST 713	100%	100%	100%	100%
CM32	*Glutamicibacter halophytocola* (MT533909.1) - 96.24%	0%	0%	0%	0%
B21A	*Glutamicibacter mishrai* (CP032549.1) - 99%	0%	0%	0%	0%
CM43	*Glutamicibacter* sp. (MK841094.1) - 97.99%	0%	0%	0%	0%
CM45	*Glutamicibacter* sp. (MK847918.1) - 98.04%	0%	0%	0%	0%
B15Y	*Glutamicibacter* sp. (MW627371.1) - 100%	0%	0%	0%	0%
EM1	*Bacillus algicola* (MN746261.1) - 96.45%	0%	0%	0%	0%
EM7	*Bacillus firmus* (KY316472.1) - 99.33%	0%	0%	0%	0%
EM8	*B. licheniformis* (JX041900.1) - 75.69%	0%	0%	0%	0%
EM2	*Bacillus* sp. (FN646629.1) - 97.80%	0%	0%	0%	0%
EM34	*Bacillus* sp. (GQ199751.1) - 96.04%	0%	0%	0%	0%
B20A	*Bacillus* sp. (KM242479.1) - 95.80%	0%	0%	0%	0%

***Score: 1 – Antimicrobial activity observed when confronted to mycoparasites; 0 – No antimicrobial activity observed.**

Supplementary Table S9: Summary of genomic data for *B. velezensis* casing isolates compared to strain QST713 (BLASTn (Nucleotide BLAST); BLASTp (Protein-Protein BLAST).

	QST713	CM5	CM19	CM35	EM5	EM39

**Similarity to [1]QST713 (%)**	100	99.99	99.99	99.99	99.99	98.38
**Genes (total)**	4,238	4,256	4,255	4,256	4,256	3,906
**Gc content**	46	45.84	46	45.85	45.84	46.43
**Genome size**	4,233,757	4,240819	4,240,819	4,240,818	4,240,818	3,929,792
**[2]CDSs (total)**	4,047	4,137	4,136	4,137	4,137	3,788
**Genes (coding)**	4,047	4,049	4,048	4,048	4,048	3,686
**CDSs (with protein)**	4,047	4,049	4,048	4,048	4,048	3,686
**Genes (RNA)**	107	119	119	119	119	118
**[3]rRNAs (5S, 16S, 23S)**	9, 8, 8	10, 9, 9	10, 9, 9	10, 9, 9	10, 9, 9	9, 9, 9
**Complete rRNAs (5S, 16S, 23S)**	9, 8, 8	10, 9, 9	10, 9, 9	10, 9, 9	10, 9, 9	9, 9, 9
**[4]tRNAs**	77	86	86	86	86	86
**[5]ncRNAs**	5	5	5	5	5	5
**Pseudo genes (total)**	84	88	88	89	89	102
**CDSs (without protein)**	84	88	88	89	89	102
**Pseudo genes (ambiguous residues)**	0 of 84	0 of 88	0 of 88	0 of 89	0 of 89	0 of 102
**Pseudo genes (frameshifted)**	48 of 84	51 of 88	51 of 88	52 of 89	52 of 89	61 of 102
**Pseudo genes (incomplete)**	58 of 84	61 of 88	61 of 88	61 of 89	61 of 89	65 of 102
**Pseudo genes (internal stop)**	14 of 84	11 of 88	11 of 88	11 of 89	11 of 89	9 of 102
**Pseudo genes (multiple problems)**	30 of 84	29 of 88	29 of 88	29 of 89	29 of 89	30 of 102

[1]Results for all strains are from analyses using NCBI PGAAP (Tatusova et al. 2016). Quality assessment of *B. velezensis* genomes using QUAST can be found in Supplementary Table S3; [2]CDS: Coding sequences; [3]rRNA: ribosomal RNA; [4]tRNA: transfer RNA; [5]ncRNA: non-coding RNA.

Supplementary Table S10: Genes in novel strains of *B.* velezensis strains containing SNPs which affect amino acid sequences compared to strain QST713. Values indicate the number of nucleotide mismatches relative to *B. velezensis* QST713, with the number of gap openings in the BLAST alignment shown in parentheses.

Locus tag	Gene	Product	BLAST mismatches (BLAST gap opens)				Amino acid differences[1]
			CM19	CM35	CM5	EM5	
BVQ_00080	*serS*	Seryl-tRNA synthetase	1 (0)	1 (0)	1 (0)	1 (0)	Sub 188: E>K
BVQ_01790	*srfAA*	Surfactin synthetase	1 (0)	1 (0)	1 (0)	1 (0)	No differences
BVQ_01795			27 (0)	27 (0)	27 (0)	27 (0)	Sub 2822: V>I; Ins 2842-2842: E; Sub 2986: A>T; Sub 2989: A>S; Sub 3025: A>E
BVQ_04115			0 (2)	0 (2)	0 (2)	0 (2)	Ins 1454: TGPTGSTGSTGETGTTGSTGATG. Del 1757: TGATGV
BVQ_04120			2 (0)				Sub 413: V>I

BVQ_06715			21 (4)	20 (4)	20 (4)	20 (4)	Sub 1014: V>I; Sub 1020: G>S; Sub 1024: S>E; Sub 1025: T>N; Sub 1028: G>T; Sub 1031: K>S
BVQ_07310				0 (1)		0 (1)	Sub 133: P>T; Sub 134: K>E
BVQ_08470	*topA*	DNA topoisomerase I		1 (0)			Sub 620: L>P
BVQ_09135	*nrdF*	β Subunit of class Ib ribonucleotide reductase (RNR)	20 (0)	20 (0)	20 (0)	20 (0)	Sub 47: L>F; Sub 49: K>T; Sub 50: N>K
BVQ_09750			7 (0)	7 (0)	7 (0)	7 (0)	Sub 2141: A>S; Sub 2180: C>G
BVQ_09755			60 (0)	60 (0)	60 (0)	60 (0)	Sub 546: E>D; Sub 691: A>S; Sub 698: A>G; Sub 717: A>E; Sub 753: V>M; Sub 797: L>F; Sub 836: N>D; Sub 901: T>N; Sub 905: E>D; Sub 930: A>V; Sub 940: M>V; Sub 1277: R>G
BVQ_09765			39 (0)	38 (0)	38 (0)	38 (0)	Sub 561: S>A; Sub 568: P>S; Sub 584: S>G; Sub 593: E>D; Sub 597: A>S; Sub 961: R>Q; Sub 1139: P>A; Sub 1147: C>G; Sub 1272: R>G
BVQ_09930			12 (0)	12 (0)	12 (0)	12 (0)	Sub 372: I>V; Sub 375: A>V
BVQ_10235			2 (0)				Sub 72: L>I
BVQ_10270				1 (0)			Sub 193: G>D
BVQ_11585	*yunB*	Sporulation protein YunB	16 (0)	16 (0)	16 (0)	16 (0)	Sub 15: M>I; Sub 105: V>A; Sub 117: G>D
BVQ_11600			3 (0)	3 (0)	3 (0)	3 (0)	No differences
BVQ_12510			16 (0)	16 (0)	16 (0)	16 (0)	Sub 3134: V>I; Sub 3155: F>V; Sub 3161: S>F; Sub 3169: I>V; Sub 3180: E>D; Sub 3199: S>P; Sub 3204: G>D
BVQ_12525			5 (0)	5 (0)	5 (0)	5 (0)	Sub 3005: D>G; Sub 3037: C>G; Sub 3110: D>G; Sub 3231: A>T
BVQ_13970			5 (0)	5 (0)	5 (0)	5 (0)	No differences
BVQ_14470			7 (0)	7 (0)	7 (0)	7 (0)	Sub 3: R>G; Sub 9: S>C ; Sub 14: G>R; Sub 28: R>G; Sub 53: F>S; Sub 53: F>S; Sub 55: G>R; Sub 62: T>A;
BVQ_16310			1 (0)	1 (0)	1 (0)	1 (0)	Sub 170: R>H
BVQ_17710			1 (0)				Sub 526: N>T
BVQ_18420			106 (2)	106 (2)	106 (2)	106 (2)	Sub 850: S>V; Sub 853: A>S; Sub 924: Q>K; Sub 938: G>E; Sub 939: E>G; Sub 940: T>A; Sub 941: L>Y; Sub 1030: I>L; Sub 1041: G>A; Sub 1049: N>D; Sub 1087: K>R; Sub 1091: V>A; Sub 1094: E>Q; Sub 1100: N>Q; Sub 1212: D>N; Sub 1214: D>E; Sub 1225: D>E; Sub 1230: H>P; Sub 1245: H>Y; Sub 1262: Q>E; Sub 1284: D>E; Sub 1291: H>Y; Sub 1307: N>D; Del 1312-1314: HTE; Sub 1318: H>N; Sub 1319: H>N; Sub 1376: A>T; Sub 1377: S>T; Sub 1379: G>E; Sub 1395: S>A; Sub 2274: Y>H; Sub 2291: E>Q; Sub 2313: E>D;

							Sub 2320: Y>H; Sub 2336: D>N; Ins 2341-2343: HTE; Sub 2347: N>H; Sub 2348: N>H; Sub 2833: L>P
BVQ_19105					1 (0)		No differences

[1]Sub = substitution, Ins = insertion, Del = deletion

Supplementary Table S11: Specialised metabolites specifically targeted by metabolic profiling using HPLC-HRMS, based on genome mining and antiSMASH analysis (see Figure 6 in the manuscript) that were not produced by any of the three *B. velezensis* strains in the given cultivation conditions on LB agar (i.e., < LOD (limit of detection)).

Compound name	Chemical formula	Exact mass	Chemical structure
**Butirosin A**	$C_{20}H_{39}N_5O_{12}$	541.25952	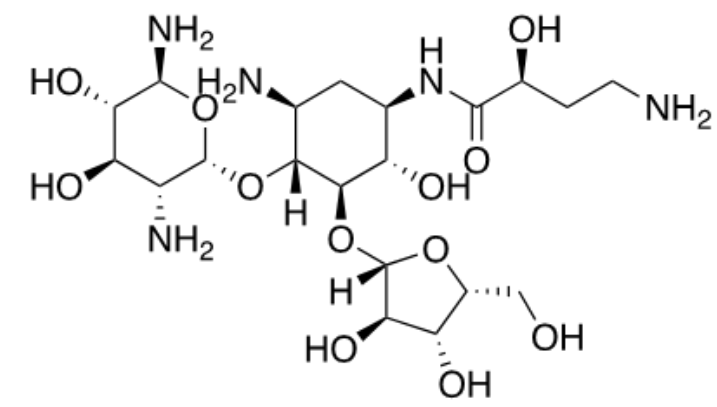
**Butirosin B**	$C_{20}H_{39}N_5O_{12}$	541.25952	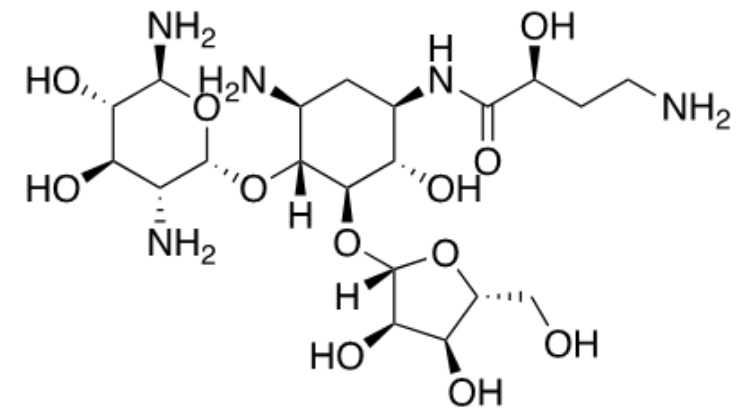
**Citrulline**	$C_6H_{13}N_3O_3$	175.09569	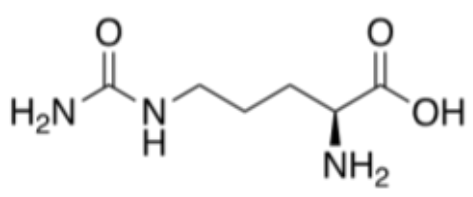
**Macrolactin A**	$C_{24}H_{34}O_5$	402.24062	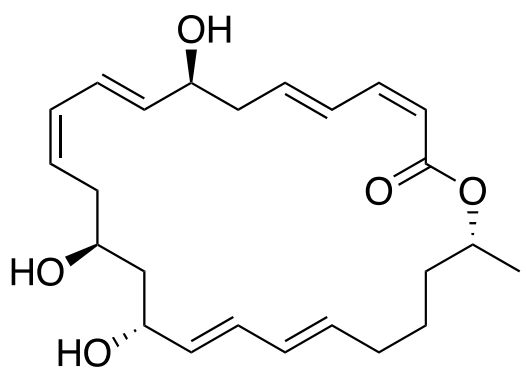
**7-*O*-Succinyl-macrolactin A**	$C_{28}H_{38}O_8$	502.25667	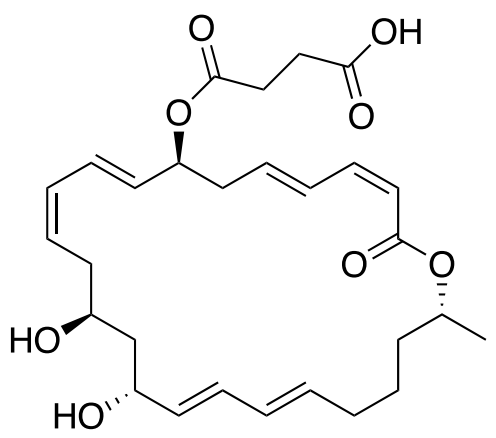
**7-*O*-Malonyl-macrolactin A**	$C_{27}H_{36}O_8$	488.24102	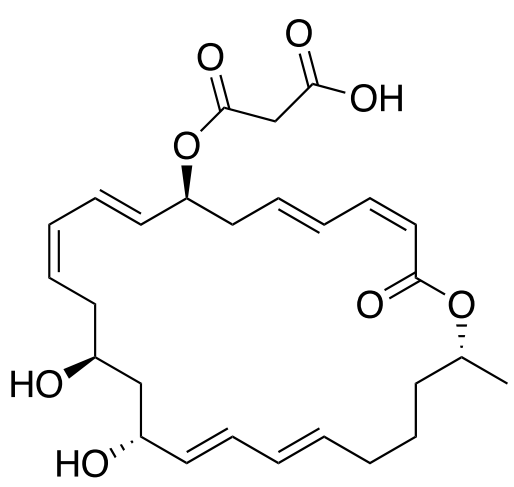

**Macrolactin B**	$C_{30}H_{44}O_{10}$	564.29345	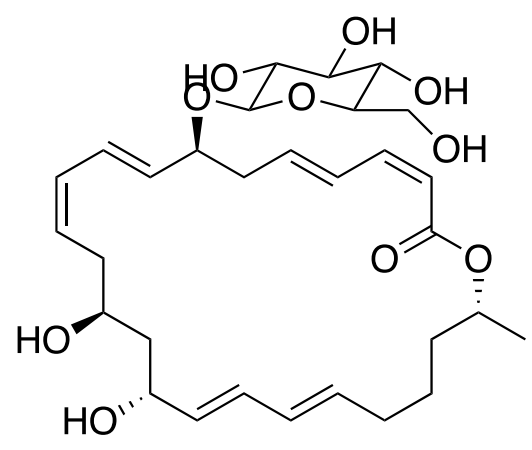
**Macrolactin C**	$C_{30}H_{44}O_{10}$	564.29345	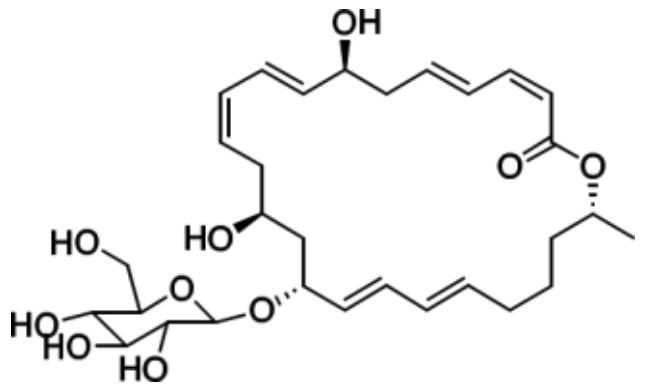
**Macrolactin D**	$C_{34}H_{48}O_{13}$	664.30949	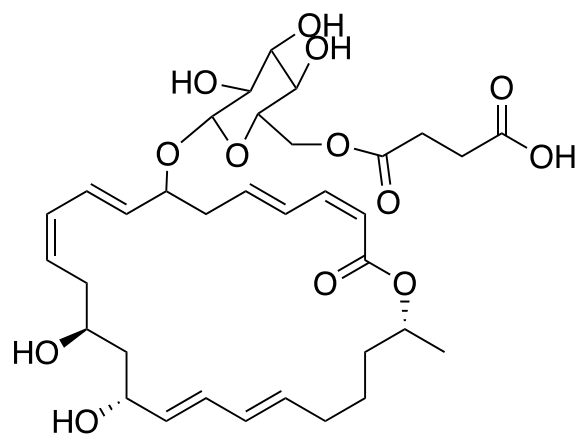
**Macrolactin E**	$C_{24}H_{32}O_5$	400.22497	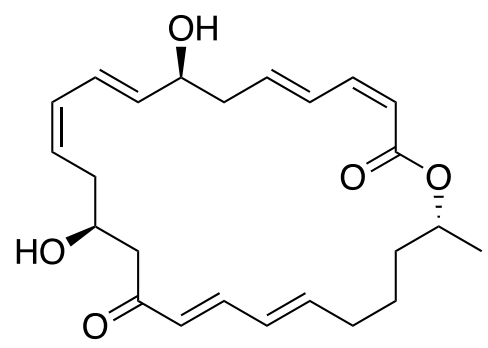
**7-*O*-Succinyl-macrolactin E**	$C_{28}H_{36}O_8$	500.24102	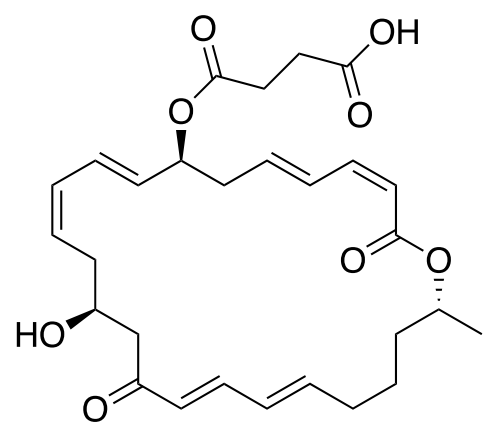
**Macrolactin F**	$C_{24}H_{34}O_5$	402.24062	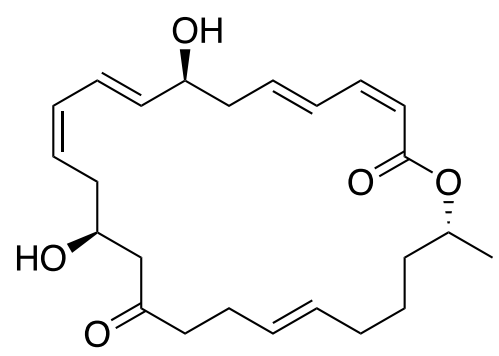
**7-*O*-Succinyl-macrolactin F**	$C_{28}H_{38}O_8$	502.25667	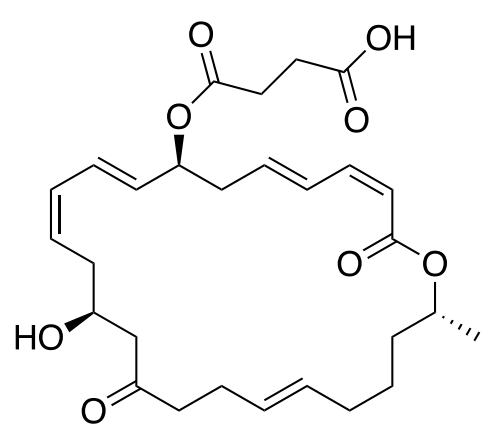
**15-Epi-dihydromacrolactin F**	$C_{24}H_{38}O_5$	406.27192	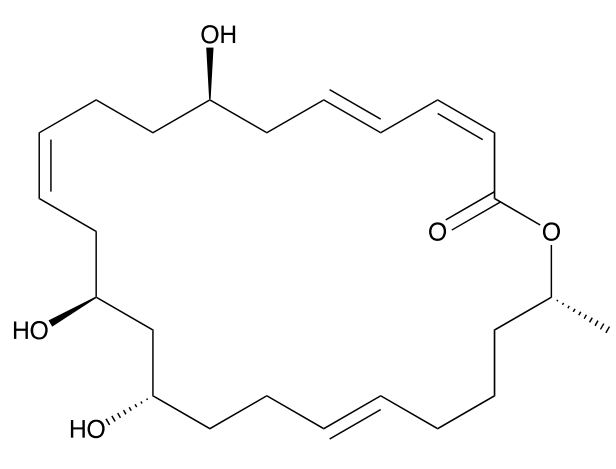

**Macrolactin G**	$C_{24}H_{34}O_5$	402.24062	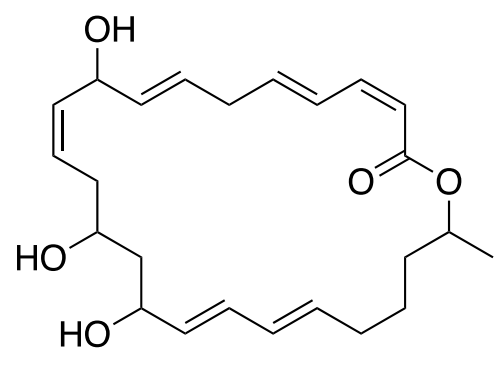
**Macrolactin H**	$C_{22}H_{32}O_5$	376.22497	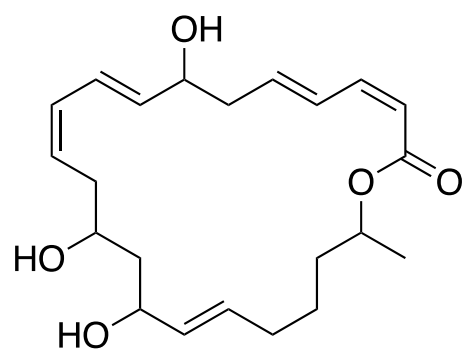
**Macrolactin I**	$C_{24}H_{34}O_5$	402.24062	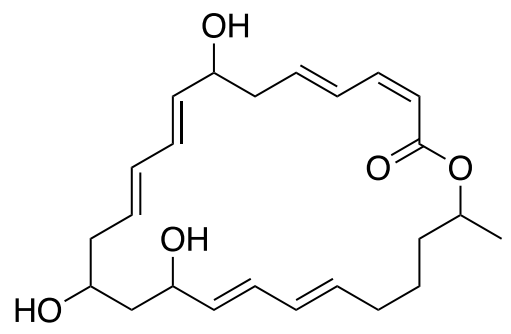
**Macrolactin J**	$C_{24}H_{34}O_5$	402.24062	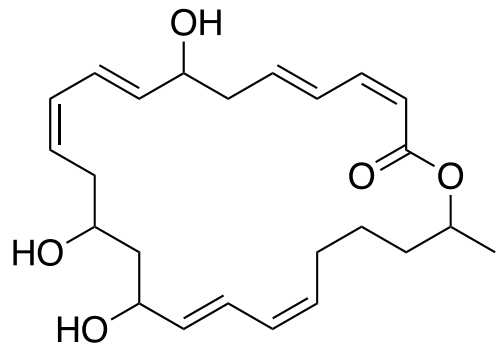
**Macrolactin K**	$C_{24}H_{34}O_5$	402.24062	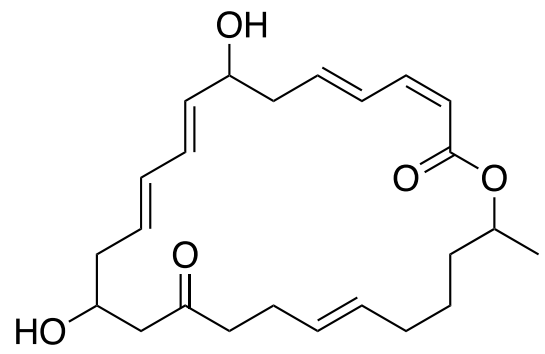
**Macrolactin L**	$C_{24}H_{34}O_5$	402.24062	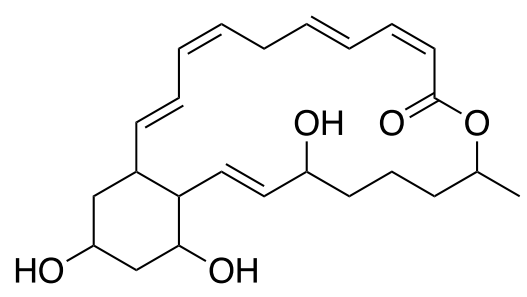
**Macrolactin M**	$C_{25}H_{36}O_5$	416.25627	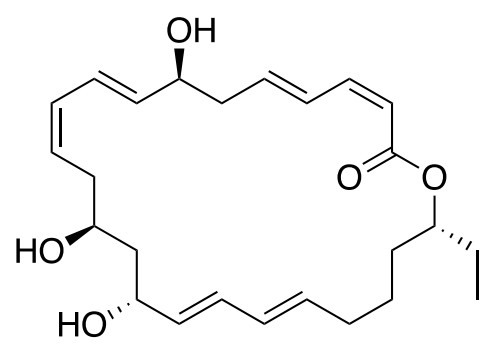
**Macrolactin N**	$C_{24}H_{34}O_4$	386.24571	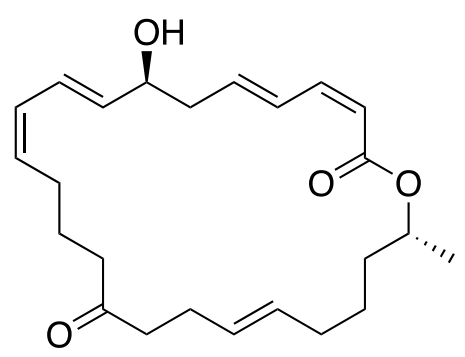
**Macrolactin O**	$C_{30}H_{44}O_{10}$	564.29345	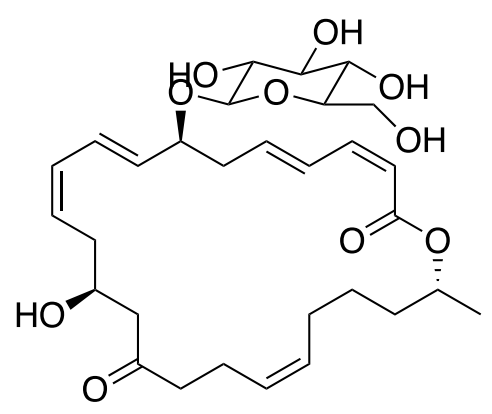

**Macrolactin P**	$C_{31}H_{46}O_{10}$	578.30910	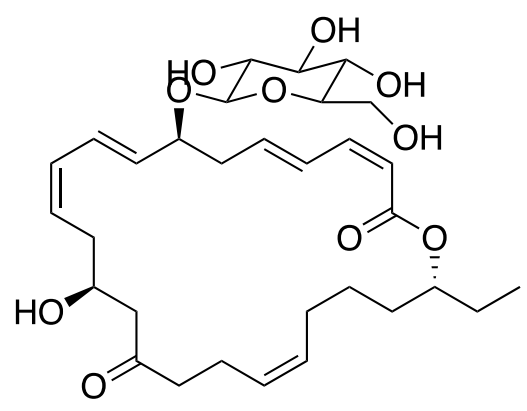
**Macrolactin Q**	$C_{30}H_{44}O_{10}$	564.29345	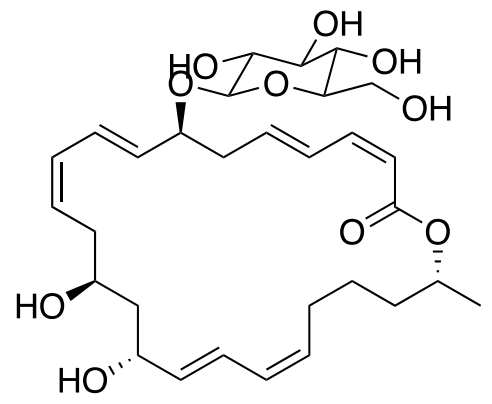
**Macrolactin R**	$C_{30}H_{44}O_{10}$	564.29345	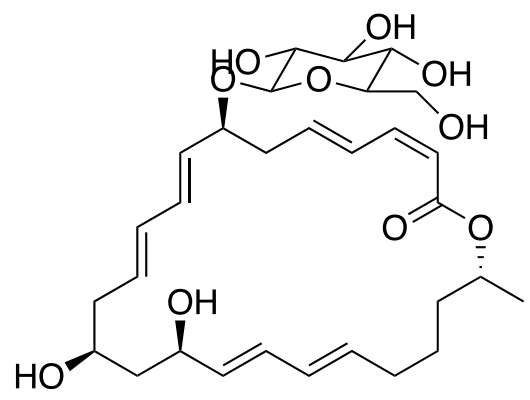
**Macrolactin S**	$C_{24}H_{34}O_{6}$	418.23554	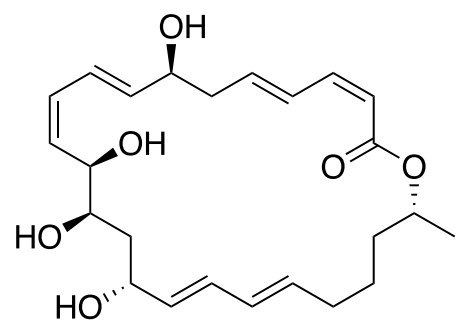
**Macrolactin T**	$C_{24}H_{38}O_{6}$	422.26684	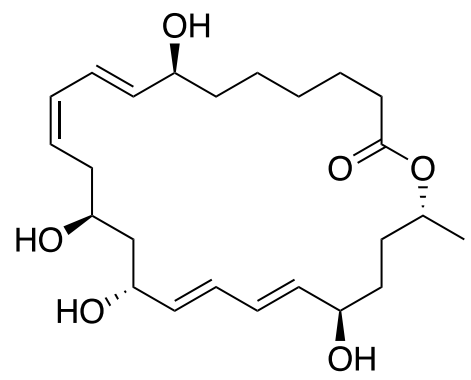
**Macrolactin U**	$C_{31}H_{44}O_{4}$	480.32396	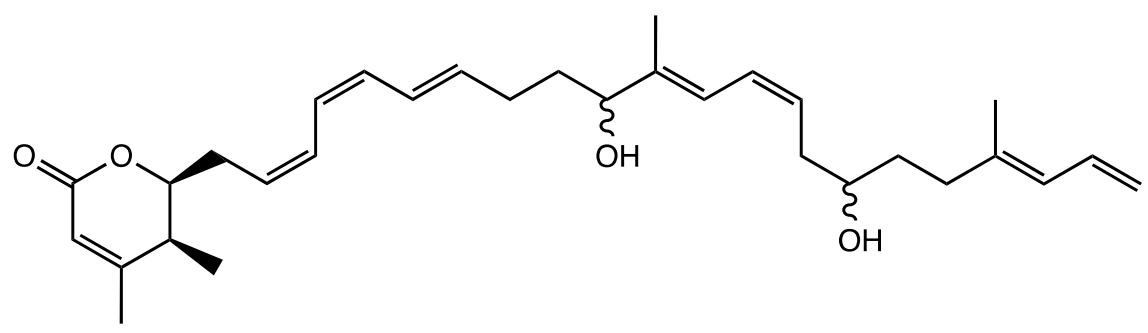
**Macrolactin X**	$C_{24}H_{34}O_{6}$	418.23554	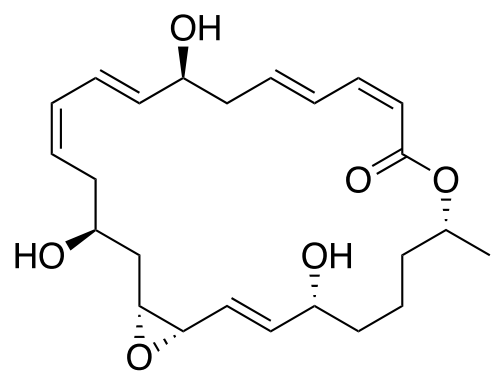
**Macrolactin Y**	$C_{25}H_{38}O_{7}$	450.26175	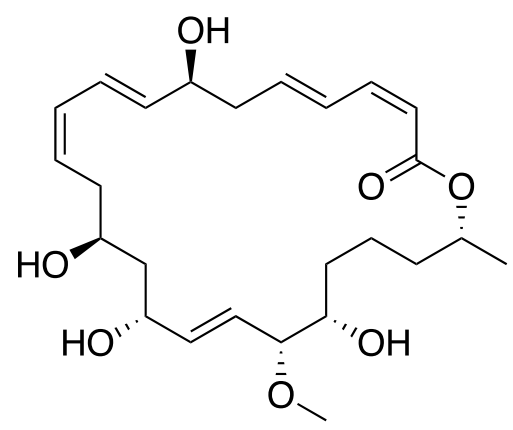

**Macrolactin Z**	$C_{29}H_{40}O_8$	516.2723 2	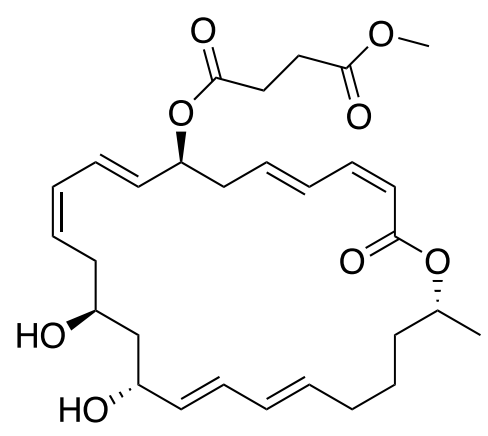
**7-*o*-Methyl-5-hydroxy-3-heptonate-macrolactin**	$C_{31}H_{48}O_7$	532.340	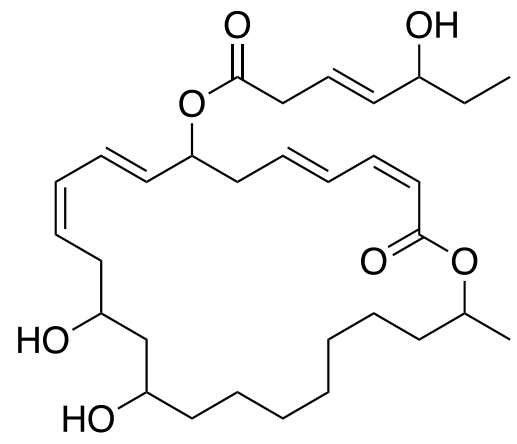

Supplementary Figures

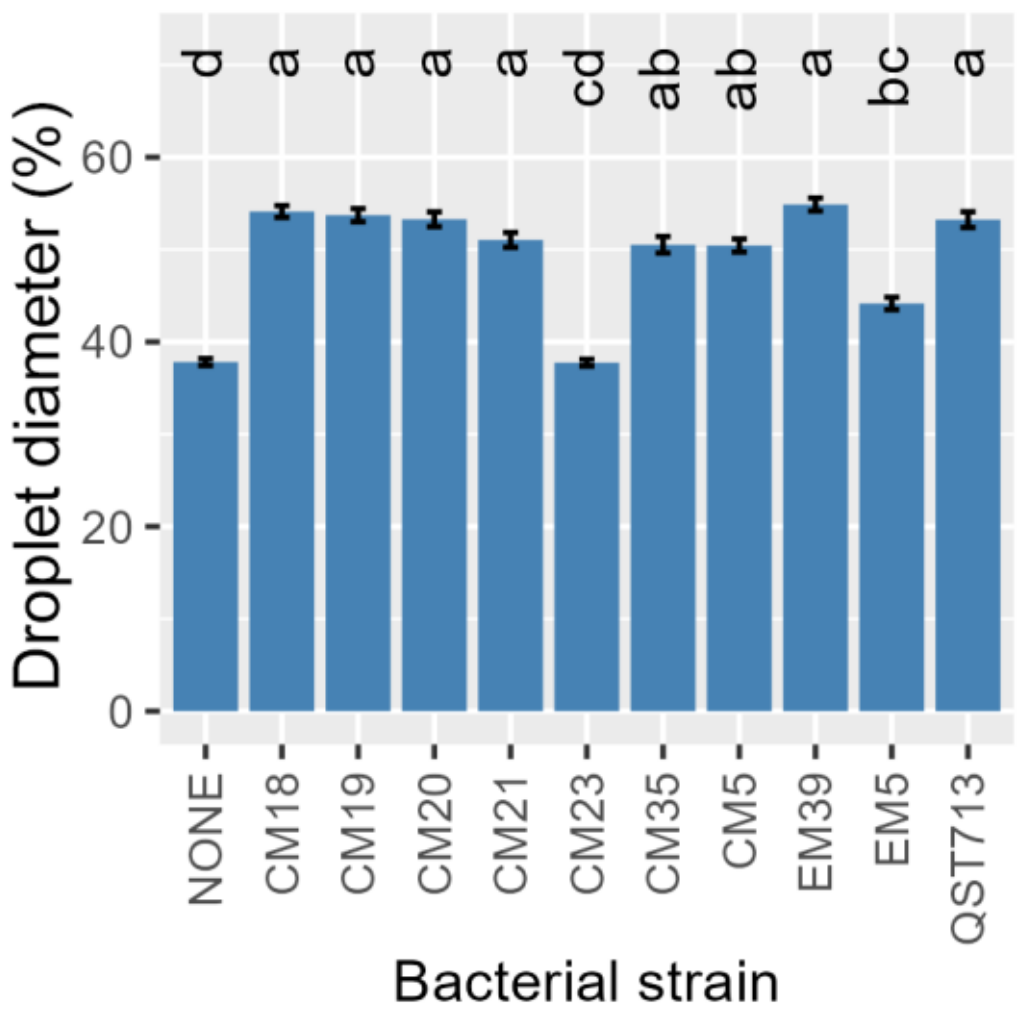

Supplementary Figure S1: Surfactant production in *B. velezensis* strains. Data shown is the mean droplet diameter of supernatant from overnight LB cultures. Droplet diameters were measured 2 minutes after pipetting onto microscope slides. Graph shows the mean and standard error of five experiments, each containing five droplets per treatment. Letters indicate groups of statistically different conditions (one-way ANOVA and Tukey's HSD test α = 0.05).

Supplementary References

Draganic V, Lozo J, Biocanin M, Dimkic I, Garalejic E, Fira D, Stankovic S, Beric T (2017) Genotyping of *Bacillus* spp. isolate collection from natural samples. Genetika-Belgrade 49(2):445-456

Fajardo-Cavazos P, Leehan JD, Nicholson WL (2018) Alterations in the spectrum of spontaneous rifampicin-resistance mutations in the *Bacillus subtilis rpo*B gene after

cultivation in the human spaceflight environment. Front Microbiol 9:192 https://doi.org/10.3389/fmicb.2018.00192

Köhl J, Vlaswinkel M, Groenenboom-de Haas B, Kastelein P, Van Hoof R, Van der Wolf J, Krijger M (2011) Survival of pathogens of Brussels sprouts (*Brassica oleracea Gemmifera* Group) in crop residues. Plant Pathol 60(4):661-670 https://doi.org/10.1111/j.1365-3059.2010.02422.x

Taguchi H, Senoura T, Hamada S, Matsui H, Kobayashi Y, Watanabe J, Wasaki J, Ito S (2008) Cloning and sequencing of the gene for cellobiose 2-epimerase from a ruminal strain of *Eubacterium cellulosolvens*. FEMS Microbiol Lett 287(1):34-40 https://doi.org/10.1111/j.1574-6968.2008.01281.x

Taparia T, Krijger M, Hodgetts J, Hendriks M, Elphinstone JG, Van der Wolf J (2020) Six multiplex TaqManTM-qPCR assays for quantitative diagnostics of *pseudomonas* species causative of bacterial blotch diseases of mushrooms. Front Microbiol 11:989 https://doi.org/10.3389/fmicb.2020.00989
